# Topographical Distribution of Neuroanatomical Abnormalities Following COVID-19 Invasion: A Systematic Literature Review

**DOI:** 10.1007/s00062-023-01344-5

**Published:** 2023-09-11

**Authors:** Ceyda Kiyak, Ogochukwu Ann Ijezie, Joseph A. Ackah, Matthew Armstrong, Jake Cowen, Deniz Cetinkaya, Hana Burianová, Theophilus N. Akudjedu

**Affiliations:** 1 Faculty of Science and Technology, Bournemouth University, Bournemouth, UK; 2 School of Psychology, University of East Anglia, Norwich, UK; 3 Institute of Medical Imaging and Visualisation, Faculty of Health and Social Sciences, Bournemouth University, 8 8GP Bournemouth, UK; 4 Department of Rehabilitation & Sports Sciences, Faculty of Health and Social Sciences, Bournemouth University, Bournemouth, UK; 5 Department of Radiology, Queen Alexandra Hospital, Portsmouth Hospitals University NHS Trust, Portsmouth, UK

**Keywords:** Brain, Spine, Coronavirus, SARS-CoV‑2, Neuroimaging

## Abstract

**Purpose:**

This systematic review is aimed at synthesising the literature base to date on the frequency and topographical distribution of neuroanatomical changes seen on imaging following COVID-19 invasion with a focus on both the acute and chronic phases of the disease.

**Methods:**

In this study, 8 databases were systematically searched to identify relevant articles published from December 2019 to March 2022 and supplemented with a manual reference search. Data were extracted from the included studies and narrative synthesis was employed to integrate the findings.

**Results:**

A total of 110 studies met the inclusion criteria and comprised 119,307 participants (including 31,073 acute and 143 long COVID-19 patients manifesting neurological alterations) and controls. Considerable variability in both the localisation and nature of neuroanatomical abnormalities are noted along the continuum with a wide range of neuropathologies relating to the cerebrovascular/neurovascular system, (sub)cortical structures (including deep grey and white matter structures), brainstem, and predominant regional and/or global alterations in the cerebellum with varying degrees of spinal involvement.

**Conclusion:**

Structural regional alterations on neuroimaging are frequently demonstrated in both the acute and chronic phases of SARS-CoV‑2 infection, particularly prevalent across subcortical, prefrontal/frontal and cortico-limbic brain areas as well as the cerebrovascular/neurovascular system. These findings contribute to our understanding of the acute and chronic effects of the virus on the nervous system and has the potential to provide information on acute and long-term treatment and neurorehabilitation decisions.

**Supplementary Information:**

The online version of this article (10.1007/s00062-023-01344-5) contains supplementary material, which is available to authorized users.

## Introduction

The typical clinical spectrum of SARS-CoV‑2 (COVID-19) infection is widespread and encompasses asymptomatic infection, mild upper and/or lower respiratory tract illness, fever, severe viral pneumonia with respiratory failure and, in some cases, death [1]. While it was initially identified as predominantly a respiratory infection [2], COVID-19 is now widely considered a multisystemic disease, causing cardiovascular, renal, gastrointestinal, hepatic, haematological [3] and metabolic disorders [4]. Accumulating evidence has highlighted potential relationships and involvement of the central nervous system (CNS) in the invasion mechanism of the virus [5, 6] as evidenced in large cohorts of patients displaying neurological manifestations [7–9]. For example, in a cohort of patients hospitalised with COVID-19 (*n* = 214) in Wuhan, 36.4% presented with neurological symptoms, including dizziness, headache, impaired consciousness, and acute cerebrovascular events. Similarly, LaRovere et al. [10] in a large retrospective study (*n* = 1695) from the USA, reported several neurological complications, including loss of taste and smell, altered awareness or confusion, fatigue/weakness, headache, and seizures or status epilepticus across a large proportion (21.5%) of the cohort.

Neuroimaging studies implicated various brain regions including the involvement of the olfactory areas coupled with prefrontal and cortico-limbic structures in the pathophysiology of COVID-19 to explain these neurological manifestations [11–13]. A recent review of several clinical case studies also highlighted spinal involvement of COVID-19 infections, providing valuable insights into the diagnoses and management of affected patients [150]. Similar findings were recently reported in previous systematic reviews [7, 14, 151]; however, most of these studies are limited by methodological heterogeneities including the inclusion of relatively small sample studies and cases (*n* < 10 patients in brain studies) and irreproducible literature search strategies.

Considering the quickly evolving nature of the pandemic and mutations of the COVID-19, it is critical to comprehensively analyse the available literature to update the whole continuum of neuroanatomical (brain and spine) imaging findings relating to all phases (i.e., acute and chronic) of the disease. This systematic review aims to collate early evidence, frequency of occurrence and topographical distribution of neuroanatomical abnormalities following COVID-19 infection with a focus on acute and chronic (including possible long COVID) disease phases. The findings will provide valuable insights into expected topographical neuroimaging features post-COVID-19 infection, and possibly guide future neurological management of patients, while adding to the evolving literature base on the long-term effects of COVID-19.

## Methods

### Protocol and Registration

The updated version of the Preferred Reporting Items for Systematic Reviews and Meta-analyses (PRISMA) guidelines [15] was employed for this study. The study protocol was registered with the International Prospective Register of Systematic Reviews (PROSPERO ID: CRD42022315428) prior to the start of the study.

### Search Strategy

The keywords required for the search were identified using the Participants, Interventions, Comparators, Outcomes, and Study (PICOS) design framework [16, 17] to guide the search and obtain the specific studies that are appropriate for the review. The terms were developed by the research team together with an expert librarian (JH) who confirmed it to be appropriate. The systematic search was conducted independently by two reviewers (CK and OAI) across key databases: PubMed (via Ovid), Scopus, ScienceDirect, EMBASE (via Ovid), PsycINFO (via Ovid), the Cochrane Library, Web of Science and CINAHL to identify relevant articles published between December 2019 and March 2022. These timepoints were selected according to the COVID-19 epidemiological trends to date [18]. The search was independently updated by two reviewers (JAA and TNA) in July 2023. The reference lists of the selected articles were hand-searched for additional studies not identified in the initial electronic search. An iterative process using controlled vocabulary, free text, synonyms, and related terms interconnected by Boolean operators (“AND” and “OR” only) was employed for the query search development. The search was conducted using the keyword combinations: COVID-19, neuroimaging, brain changes and spinal changes (Table 1).

**Table 1:** Strategy employed for searching relevant articles

Search term	Related keywords or terms
COVID-19	COVID-19 OR coronavirus OR 2019-ncov OR sars-cov‑2 OR cov-19 OR covid OR HCoV OR severe acute respiratory syndrome coronavirus 2 OR C‑19 OR coronavirus disease 2019
Neuroimaging	Neuroanatom* OR neuro-anatom* OR Neuroradiolog* OR Neuro-radiolog* OR neuroimag* OR neuro-imag* OR MRI OR magnetic resonance imag* OR CT OR comput* tomography OR medical imag*
Brain changes	Neurolog* OR brain OR central nervous system OR CNS OR encephalo* OR abnormal* OR anomal* OR deform*
Spinal changes	Neurolog* OR spine OR central nervous system OR CNS OR myelitis* OR abnormal* OR anomal* OR deform*

### Eligibility Criteria

In accordance with the PICOS framework, the eligibility criteria are detailed:

i) Study design: case-control studies, observational cohort studies (retrospective and prospective studies), and randomised controlled trials (RCTs) were included. Case studies/series that specifically focused on the brain were included if they had a sample size ≥ 10. Considering the rarity of studies focusing on spinal changes, studies of sample size ≥ 5 were included.
ii) Participants: the participants were patients with acute (or current) and long (or post) COVID-19 disease. There were no restrictions regarding age, sex, ethnicity and/or disease risk groups with a COVID-19 infection. Studies reporting on patients with COVID-19 without neuroimaging and/or neurological data were excluded.
iii) Interventions: interventional and/or follow-up studies reporting structural and functional neuroanatomical changes using neuroimaging i.e., magnetic resonance imaging (MRI) and computed tomography (CT) in COVID-19 patients were included.
iv) Comparators: studies reporting associations between clinical symptomatology and observed neuroanatomical changes were included.
v) Outcomes: studies were eligible whose main outcomes were localisation of structural and functional neuroanatomical changes in patients with COVID-19, measured using neuroimaging methods, such as MRI and CT scans, and clinical symptomatology (including neurological and/or psychological measures) in the patients. Studies reporting changes in the brain and the spine (and associated structures) measured via CT scan, structural and/or functional magnetic resonance imaging (fMRI) scan, and hybrid imaging (e.g., positron emission tomography-computed tomography (PET-CT) after COVID-19 infection were included. Studies were also considered if they reported multiple diagnoses (e.g., both brain, spinal and other related clinical conditions), but data on the changes in brain and spinal structure or activity were explicitly collected and analysed separately. Studies employing other neuroimaging modalities, such as electroencephalogram (EEG), were excluded as the study focussed on structural measures of neuroanatomy.

In addition to the PICOS framework requirements, inclusion was limited to only articles published in English. Review articles, pictorial essays, letters to the editor, correspondence, postscript and research letters, unpublished data, commentaries, opinion papers, thesis/dissertations, conference abstracts, and other topical proceedings were excluded.

### Study Selection

In the first phase of screening, two reviewers (CK and OAI) independently screened the titles and abstracts to exclude articles that were irrelevant to the systematic review. The second phase related to independent full text screening of the remaining articles that met the inclusion criteria. Disagreements were resolved by consensus and/or by consultation with the principal investigator (TNA). The screening process was undertaken using the web-based version of the Rayyan software [19].

### Data Extraction

Data were collected manually via a tabular template for relevant information and recorded in Microsoft Excel 365 (Microsoft Inc, Redmond, WA, USA). The following characteristics were extracted: references and country of origin, study type, total number of participants, total number of acute and long COVID-19 patients, neuroimaging modality, neuroanatomical regions involved, and clinical findings. Two reviewers (CK and OAI) independently extracted data from the included studies and disagreements were resolved in a consensus meeting with the principal investigator (TNA).

### Risk of Bias and Quality Assessment

Bias of the included studies was assessed by two independent reviewers (CK and OAI) using the Risk of Bias Assessment Tool for Nonrandomised Studies (RoBANS) [20]. Data were extracted and input into a Microsoft Excel spreadsheet by each reviewer and classified into three grades: low risk, high risk, or unclear. The outcome was evaluated by a third reviewer (JAA) and the reported discrepancies were resolved through discussion or through consultation with the research team in a consensus meeting.

## Results

### Literature Search Outcome and Management

**Fig. 1:**
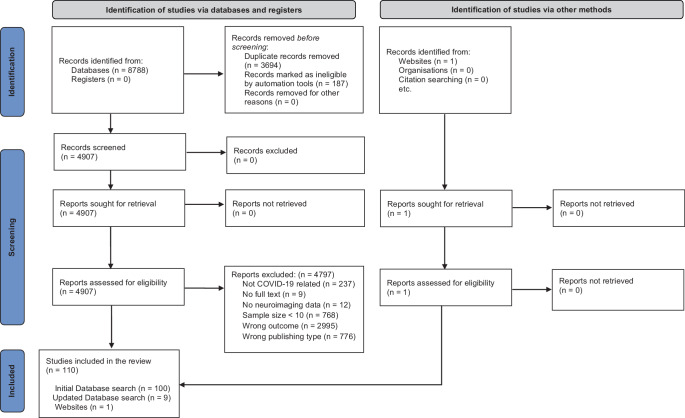
PRISMA flowchart showing study identification, screening, selection process, and eligibility criteria for the included articles

A PRISMA flowchart briefly describing the article identification, screening, and selection process is detailed (Fig. 1). A total of 8788 articles were identified through database searches. Of these, a total of 4907 remained after removal of duplicates at the end of the identification phase. Following the application of the inclusion and exclusion criteria during full text check, 4797 articles were excluded. Additional searches on ResearchGate and Google Scholar were performed for a complete list and 1 additional article was identified. The updated search included 9 relevant articles. A total of 110 articles were eligible and included for this review (Fig. 1).

### Characteristics of the Included Studies

Characteristics of the included studies are briefly presented in Table 2 and in supplementary material 1. Of the 110 included studies, 76 (69.09%) were retrospective, 20 (18.18%) were prospective, 8 (7.27%) were cross-sectional, and 5 (4.55%) were observational.

**Table 2:** Summary characteristics of the included studies

S/*N*	Author, year and location	Study type	Total number of participants	Total number of acute COVID-19 patients	Total number of long COVID-19 patients	Neuroimaging modality	Brain regions implicated																Spinal changes
							Grey matter													White matter		Cerebro/neurovascular system	
							Cortical regions								Subcortical regions					Corpus callosum	Other tracts		
							OFL	FL	MC	SC	PL	TL	OL	OR	Olfactory	Amygdala	BG and T	Cerebellum	Brainstem				
1	Abdelzaher et al., 2022, Egypt [21]	RS	135	135	N/A	CT					X						X						
2	Abenza-Abildúa et al., 2020, Spain [22]	RS	54	30	N/A	CT and MRI					X											X	
3	Agarwal et al., 2020, USA [23]	RS	115	115	N/A	MRI					x							x		x	x	x	
4	Agarwal et al., 2021, USA [24]	RS	4530	21	N/A	MRI												X	X				
5	Al-Mufti et al., 2021, USA [25]	RS	13500	13500	N/A	CT and MRI						x	x										
6	Alonazi et al., 2021, Saudi Arabia [26]	RS	62	46	N/A	CT and MRI					x		x				x						
7	Altunisik et al., 2021, Turkey [27]	RS	116	36	N/A	MRI		X							X								
8	Alves et al., 2021, Brazil [28]	RS	29	27	N/A	CT		x			x	x					x	x				x	
9	Aragao et al., 2021, Brazil [29]	RS	232	232	N/A	CT and MRI				x	x				x			x		x			
10	Arandela et al., 2021, USA [30]	RS	10	10	N/A	CT and MRI		X			X		X					X	X			X	
11	Arica-Polat et al., 2022, Turkey [31]	CS	176	170	N/A	MRI		x			x												
12	Azab et al., 2021, Egypt [32]	RS	1500	970	N/A	CT and MRI															X	X	
13	Bruce et al., 2021, USA [33]	RS	12	12	N/A	CT											X					X	
14	Bungenberg et al., 2022, Germany [34]	CS	50	50	N/A	MRI	X	X				X			X		X			X			
15	Burulday et al., 2022, Turkey [35]	RS	23	23	N/A	MRI									X	X							
16	Büttner et al., 2021, Germany [36]	RS	565	34	N/A	CT and MRI															X		
17	Chammas et al., 2021, France [37]	RS	112	112	19	MRI														x	x		
18	Chougar et al., 2020, France [38]	CS	1176	73	N/A	MRI											X			X	X	X	
19	Conklin et al., 2021, USA [39]	RS	154	16	N/A	CT and MRI												X		X	X		
20	Coolen et al., 2020, Belgium [40]	PS	62	19 (post-mortem)	N/A	MRI	x										x						
21	D’Amore et al., 2020, Italy [41]	OB	27	15	N/A	CT and MRI		X			X		X								X	X	
22	Deeb et al., 2021, UAE [42]	RS	1075	33	N/A	CT and MRI		X			X		X					X			X	X	
23	Delorme et al., 2021, France [43]	RS	1979	245	N/A	MRI											X				X	X	
24	Dilber et al., 2021, Turkey [44]	RS	2530	382	N/A	MRI																	
25	Dixon et al., 2020, UK [45]	RS	30	10	N/A	MRI														x			
26	Dodd et al., 2021, USA [46]	RS	10	10	N/A	CT		x															
27	Douaud et al., 2022, UK [11]	RS	785	401	N/A	MRI	x	x			x		x			x							
28	Duan et al., 2021, USA [47]	OB	120	58	N/A	CT						x						x		x		x	
29	Elizondo et al., 2021, Mexico [48]	RS	481	47	N/A	MRI		x							x		x	x			x	x	
30	Ermis et al., 2021, Germany [49]	PS	53	53	N/A	CT and MRI		X			X						X			X	X		
31	Escalard et al., 2020, France [50]	PS	12	12	N/A	CT and MRI							x					x				x	
32	Eskandar et al., 2021, USA [51]	RS	4711	581	N/A	CT and MRI																X	
33	Fällmar et al., 2021, Sweden [52]	PS	19	19	N/A	MRI		X				X					X	X	X	X	X		
34	Flores-Silva et al., 2021, Mexico [53]	PS	1072	163	N/A	CT and MRI		X			X		X									X	
35	Franceschi et al., 2020, USA [54]	OB	10	10	N/A	CT and MRI		X			X	X	X	x			X	X			X		
36	Freeman et al., 2021, USA [55]	RS	2820	59	N/A	MRI												x		x		x	
37	Garcia et al., 2021, USA [56]	CS	18	18	N/A	MRI	x	x			x							x					
38	García-Azorín et al., 2021, Spain [57]	OB	233	221	N/A	CT and MRI					X						X			X	X	X	
39	Gogu et al., 2021, Romania [58]	RS	1866	101	N/A	CT		x			x	x	x							x			
40	Gorgulu et al., 2021, Turkey [59]	RS	1093	42	N/A	CT											x	x					
41	Greenway et al. 2021, USA [60]	RS	8675	180	N/A	CT and MRI		X			X	X	X				X	X			X		
42	Guilmot et al., 2021, Belgium [61]	PS	349	15	N/A	CT and MRI													X			X	X
43	Günbey et al., 2021, Turkey [62]	RS	381	354	N/A	CT and MRI		x			x	x					x	x		x			
44	Hazzaa, 2021, UK [63]	RS	23	23	N/A	CT and MRI						X	X				X			X	X	X	
45	Hellgren et al., 2021, Sweden [64]	OB	734	35	N/A	MRI		x		x	x										x		
46	Hernandez-Fernandez, 2020, Spain [65]	RS	1683	121	N/A	CT		x			x		x										
47	Iqbal et al., 2021, Qatar [66]	RS	15	15	N/A	CT and MRI															X		
48	Jain et al., 2020, USA [67]	RS	3218	454	N/A	CT												x		x			
49	Jegatheeswaran et al., 2022, Canada [68]	RS	422	103	N/A	CT and MRI					X								X	X	X	X	X
50	Jensen-Kondering et al., 2021, Germany [69]	RS	12	12	2	MRI											x	x			x		
51	Kalekar et al., 2021, India [70]	RS	80	80	N/A	CT and MRI		X		X	X	X	X				X	X	X		X	X	X
52	Kandemirli et al., 2021, Turkey [71]	PS	23	23	N/A	MRI							X		X							X	
53	Karvigh et al., 2021, Iran [72]	RS	508	10	N/A	CT																X	
54	Katz et al., 2020, USA [73]	RS	585	86	N/A	CT and MRI																x	
55	Keller et al., 2020, UK [74]	PS	32	8	N/A	MRI											X	X				X	
56	Kelsch et al., 2021, USA [75]	RS	671	648	N/A	CT and MRI		X			X						X	X		X	X	X	X
57	Khedr et al., 2021, Egypt [76]	RS	439	55	N/A	CT and MRI		x				x	x				x	x					X
58	Khedr et al., 2021, Egypt [77]	RS	439	439	N/A	CT and MRI									X							X	
59	Kiatkittikul et al., 2022, Thailand [78]	RS	13	N/A	13	PET/CT and PET/rsfMRI		x			x	x	x				x						
60	Klironomos et al., 2020, Sweden [79]	RS	2611	185	N/A	CT and MRI									X			X		X			X
61	Kremer et al., 2020, France [8]	RS	64	64	N/A	MRI		X				X					X	X		X	X	X	
62	Kulkarni et al., 2022, India [80]	RS	102	49	N/A	MRI		x					x					x					
63	Lambrecq et al., 2021, France [81]	RS	72	64	N/A	MRI											x	x	x	x		x	
64	LaRovere et al., 2021, USA [10]	RS	1784	1695	N/A	CT or MRI		X									X			X	X		
65	Lersy et al., 2021, France [82]	RS	80	19	N/A	MRI		x												x			
66	Lin et al., 2020, USA [83]	RS	2054	278	N/A	CT and MRI							x				x			x			
67	Lindan et al., 2021, USA [84]	RS	429	38	N/A	CT and MRI		X					X				X	X		X	X	X	X
68	Lu et al., 2020, China [85]	PS	99	60	60	MRI		x			x	x	x		x		x	x		x			
69	Mahammedi et al., 2021, USA [86]	RS	172	135	N/A	CT and MRI		x				x	x					x		x	x	x	
70	Marcic et al., 2021, Croatia [87]	CS	55	39	N/A	MRI		X															
71	Mekkawy et al., 2022, Egypt [88]	PS	582	582	N/A	CT and MRI																X	X
72	Meppiel et al., 2021, France [89]	RS	222	102	N/A	MRI						X					X	X	X			X	
73	Metwally et al., 2022, Egypt [90]	CS	63	63	N/A	MRI																	
74	Naval-Baudin et al., 2021, Spain [91]	RS	149	100	N/A	CT					x	x							x				
75	Nawabi et al., 2020, Germany [92]	RS	18	18	N/A	CT											x	x	x			x	
76	Niesen et al., 2021, Belgium [93]	PS	12	12	N/A	PET-MR	X			X					X		X	X					
77	Orman et al., 2021, USA [94]	RS	4351	20	N/A	MRI							X										
78	Palabiyik et al., 2021, Turkey [95]	RS	45	45	N/A	CT and MRI					X						X	X		X	X		X
79	Paterson et al., 2020, UK [96]	RS	43	43	N/A	CT and MRI						X	X				X	X	X		X	X	X
80	Pons-Escoda et al., 2020, Spain [97]	CS	2249	103	N/A	CT and MRI					X						X	X					
81	Qin et al., 2021, China [98]	CS	51	51	N/A	MRI						X				X					X		
82	Radmanesh et al., 2020, USA [99]	RS	3661	242	N/A	CT and MRI														x			
83	Rapalino et al., 2021, USA [100]	RS	7146	27	N/A	MRI												x		x			
84	Rehmani et al., 2021, USA [101]	RS	16	16	N/A	CT and MRI						X					X				X	X	
85	Remsik et al., 2021, USA [102]	PS	18	18	N/A	CT and MRI								X				X					
86	Rhally et al., 2021, Switzerland [103]	RS	41	20	N/A	MRI						x								x			
87	Rifino et al., 2021, Italy [104]	RS	1760	137	N/A	CT and MRI																X	X
88	Rouyer et al., 2020, France [105]	PS	13	13	N/A	MRI								x								x	
89	Sabayan et al., 2021, Iran [106]	RS	18407	15	N/A	CT																	X
90	Saleh and Shaban, 2021, Egypt [107]	PS	70	70	N/A	MRI							x							x			
91	Sandoval et al. 2021, Chile [108]	RS	90	13	N/A	CT and MRI		X				X						X			X		
92	Sawlani et al., 2021, UK [109]	RS	3403	166	N/A	CT and MRI							x				x	x		x			
93	Scullen et al., 2020, USA [110]	RS	27	27	N/A	CT							x					x					
94	Sollini et al., 2021, Italy [111]	PS	13	N/A	13	[18F] FDG-PET/CT	X							X			X		X				
95	Strauss et al., 2020, USA [112]	RS	12	12	N/A	MRI	X								X	X							
96	Triay et al., 2021, USA [113]	RS	777	32	N/A	MRI											X				X	X	
97	Tuma et al., 2021, Brazil [114]	RS	1720	66	N/A	CT								x									
98	Uginet et al., 2021, Switzerland [115]	RS	707	31	N/A	MRI														x			
99	Xiong et al., 2020, China [116]	RS	917	917	N/A	CT		X			X		X										
100	Yadav et al., 2022, India [117]	RS	133	50	N/A	CT and MRI						X											
101	Yoon et al., 2020, USA [118]	RS	641	150	N/A	CT and MRI						x	x	x				x				x	
102	Khair et al., 2022, USA [144]	PS	5	5	N/A	MRI								X		X	X	X	X			X	X
103	Avila et al., 2023, USA [145]	PS	12	12	N/A	MRI						X	X	X				X	X	X	X		X
104	Applewhite et al., 2020, USA [146]	RS	37	37	N/A	CT																	X
105	Abrams et al., 2021, USA [147]	PS	5	N/A	5	MRI																	X
106	Mahammedi et al., 2020, Italy [148]	RS	108	108	N/A	CT and MRI		X			X			X			X	X			X	X	X
107	Mehan et al., 2020, USA [149]	RS	9	9	N/A	MRI																	X
108	Dressing et al., 2022, Germany [152]	PS	31	N/A	31	MRI																	
109	Rau et al., 2022, Germany [153]	PS	20	20	N/A	MRI		x			x	x									x		
110	Faro et al., 2023 [154]	RS	4342	4342	N/A	CT and MRI																	

*BGT* basal ganglia and thalamus, *CS*_cross-sectional, *CT* computed tomography, *FL* frontal lobe, *MC* motor cortex, *MRI* magnetic resonance imaging, *N/A* not available, *PL* parietal lobe, *PET* positron emission tomography, *OB* observational, *OFL* orbitofrontal lobe, *OL* occipital lobe, *OR* other regions, *PS* prospective, *RS* retrospective, *SARS-CoV‑2* severe acute respiratory syndrome coronavirus 2, *SC* sensory cortex, *TL* temporal lobe, *UAE* United Arab Emirates, *UK* United Kingdom, *USA* United States of America

Total participants reported in the included studies amounted to 119,307 (individual study sample sizes ranged from 10 to 18,407) including 31,073 acute and 143 long COVID-19 (patients manifesting neurological alterations) and controls. There was an uneven geographical distribution of the included studies in relation to study sites with 31.19% (*n* = 34) from United States research centres (supplementary material S1); however, no differences in neuroanatomical distribution of findings in relation to the geographical sites where the included studies were conducted were found.

Of the included articles, a proportion of studies utilised the following neuroimaging modalities: 45 (40.90%) combined brain MRI and CT, 44 (40.00%) MRI only, 17 (15.45%) CT only, 1 (0.91%) PET/MR, 1 (0.91%) PET/CT and PET/resting state functional magnetic resonance imaging (rsfMRI), and 1 (0.91%) for 18F-fluorodeoxyglucose positron emission tomography-computed tomography scan (^18^ FDG-PET/CT). Of note, 16.36% (*n* = 18) of studies comprehensively explored both the spine and the brain simultaneously (Table 2; supplementary material S1).

### Risk of Bias and Quality Assessment

**Fig. 2:**
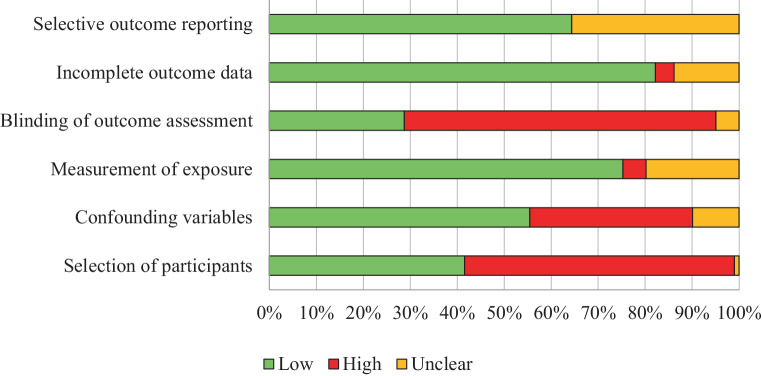
Risk of bias diagram depicting the proportion of studies with low, high, or unclear assessment across domains

Figure 2 provides an overview of the six domain outcome summaries from the risk of bias assessment. The domain relating to the selection of participants in the included studies reported 59 (53.64%) studies to be of high risk of bias and 50 (45.45%) at low risk. The domain relating to confounding variables highlighted 63 (57.27%) studies as low risk of bias. Most studies (*n* = 84, 76.36%) recorded low risk for measurement of exposure. Of note, most studies (*n* = 69, 62.73%) were considered high risk for blinding of outcome assessment. For incomplete outcome data, 92 (83.64%) studies were scored as low risk of bias. For selective outcome reporting, 73 (66.36%) studies were a low risk of bias, and the risk was unclear for 36 (32.72) articles (See Table S2).

### Neuroanatomical Changes from Acute Effects of SARS-CoV-2 Infection

There was considerable variability in both the localisation and nature of brain abnormalities, resulting in a wide range of neuropathologies. The commonly reported abnormalities identified on neuroimaging included cerebral ischemia, in the form of acute, subacute or chronic infarction, haemorrhage, acute strokes and persistent microhaemorrhages, cerebral venous sinus thrombosis, and supratentorial and infratentorial white matter changes.

Regional brain changes were reported in all the included studies. Of the included studies, a high incidence of neuropathology was identified in the cerebellum (41.6%), cerebrovascular/neurovascular system (39.6%), basal ganglia and thalamus (18.6%), corpus callosum (35.6%), frontal lobe (35.6%), parietal lobe (29.7%) and occipital lobe (29.7%). Other implicated regions included the motor cortex, orbitofrontal cortex, sensory cortex, temporal lobe, brainstem, amygdala, and several white matter tracts (i.e., deep/subcortical and/or nonspecific). Of note, alterations of the primary olfactory cortices were observed across almost all studies that reported on the acute effects of the infection.

Almost all (94.44%, *n* = 17/18) studies that reported on the spine highlighted several degrees of spinal cord involvement in the acute phase, especially in non-critical patients. However, MRI studies have reported enhancement and hyperintensity involvement in the cauda equina fibres, central cord, and nerve roots among some patients [61, 68, 70, 75, 76, 79, 86, 88, 95, 96, 104, 108, 144–146, 148, 149].

### Regional Neuroanatomical Changes in Long COVID-19

Regional brain alterations appear to persist postinfection [37, 69, 78, 85, 111]. For example, Sollini et al. [111], in an ^18^F‑FDG-PET/CT study (total sample, *n* = 13 adult long COVID-19 patients), reported alterations in multiple regional brain networks including the primary olfactory networks (5 patients, 41.7%), involving the occipital lobe (5 patients, 41.7%), and the thalamic network (1 patient, 8.3%). In a similar ^18^F‑FDG-PET/CT study (total sample, *n* = 13 post-acute COVID-19 patients), Kiatkittikul et al. [78] reported hypometabolism in the parietal lobe (11 patients, 91.7%), temporal lobe (11 patients, 91.7%), frontal lobe (5 patients, 41.7%), occipital lobe (5 patients, 41.7%), and thalamus (1 patient, 8.3%) with general recovery and/or preservation of other regional neuroanatomical structures. In contrast to these findings, Dressing et al. [152] observed no distinct pathological findings of hypermetabolic predominance.

Findings relating to the spine were mostly of either degenerative character with other observations including demyelinated plaques, and spinal lesions while others remained unremarkable despite persistence of clinical symptoms [108, 147].

### Clinical Symptomologies of Neurological Relevance Reported Across Studies and Disease Phases

Clinical findings were reported across 94 of the 101 included studies. Headache*, *a commonly reported neurological symptom of COVID-19 infection, was reported by 53 out of 101 (56.3%) of the included studies, followed less commonly by seizure (43.6%), encephalopathy (28.7%), (haemorrhage (23.6%), ischaemic infarcts (13.8%)) and associated strokes (23.4%) (Table 3).

**Table 3:** Prevalence ranking of clinical symptomatologies across disease phases

Clinical symptomologies	Acute phase	Long/chronic disease phase	% of included studies reporting incidence	Commentary
Headache	**✓**	**✓**	56.3	Headaches were reported across multiples studies over the entire course (acute and chronic) of the disease
Seizures	**✓**	**X**	43.6	These sudden and mostly uncontrollable episodes were mostly associated with the acute phase of the disease
Encephalopathy	**✓**	**X**	28.7	Encompass a series of brain dysfunctions and pathologies including altered mental state which was majorly reported across studies reporting on the acute phase of the disease
Stroke	**✓**	**X**	23.4	The COVID-related strokes reported are majorly of two types, haemorrhagic and ischaemic and were mostly associated with the acute phase of the disease

✓ = present, X = absent/not reported

Other clinical findings which were less frequently reported across the included studies are summarised (see S1 Table).

## Discussion

The findings revealed considerable variability in both the localisation and nature of abnormalities detected on neuroimaging, encompassing a wide range of neuropathologies affecting the cerebrovascular/neurovascular system, basal ganglia and thalamus, corpus callosum, motor cortex, orbitofrontal lobe, sensory cortex, temporal lobe, brainstem, amygdala and predominant regional and/or global alterations in the cerebellum. Of note, alterations of the primary olfactory cortex were observed across almost all studies that reported on the acute effects of the infection. Olfactory brain network hypometabolism in long COVID-19 patients has also been noted. Along the neuroanatomical continuum to the spine, transverse myelitis, meningoencephalitis, and various degrees of inflammatory reaction along the spinal cord were noted especially in the acute phase of the disease.

### Neuroanatomical Changes in the Acute Phase of the Disease

Of the included participants in the reported studies, 23% had acute COVID-19, presenting with neurological manifestations, and underwent either brain CT or MR imaging. SARS-CoV‑2 was associated with structural neuroanatomical [40] and intensity abnormalities [29, 79, 83, 112] in the olfactory bulb/tract. These deficits consisted of altered cortical volume [85], thickness [119] and hypometabolism [111, 120, 121]. Additional alterations of the primary olfactory cortex and related networks were observed across almost all studies that reported on the acute effects of the infection [11, 34, 40, 56, 93, 111, 112]. These findings underscore the importance of the olfactory system as a unique anatomical element that provides an optimal conduit for neuroinvasion [13]. In terms of symptomatology, the primary involvement of the olfactory system in the pathophysiology of the COVID-19 infection explains anosmia and in some cohorts headaches as an early marker of the SARS-CoV‑2 infection [122].

Surprisingly, the cerebellum was found to be affected in the acute stages of the disease and across most studies and case studies [8, 23, 24, 28–30, 39, 42, 47, 48, 50, 52, 54–56, 59, 60, 62, 67, 69, 70, 74–76, 79–81, 84–86, 89, 92, 93, 95–97, 100, 102, 108–110, 118], mostly presenting as cerebellar ataxia (for example, see [123, 124]). This was characterised by accentuation of atrophy in the cerebellum and its corresponding neural connections. SARS-CoV‑2 affects the cerebellum via direct viral invasion, but even more so through its effects on immune, haematological, and metabolic pathways [125]. The involvement of the cerebellum in the pathophysiology of COVID-19 is not fully understood; however, our findings highlight a high prevalence of involvement of this structure and calls for further investigation. Other neuroanatomical alterations were reported in acute COVID-19 across the cerebrovascular/neurovascular system, basal ganglia and thalamus, corpus callosum, regional frontal lobe, parietal lobe, and occipital lobe. The medial temporal lobe appears particularly vulnerable in the pathophysiology of COVID-19, thus resulting in cognitive deficits leading to language and memory impairments [126].

White matter abnormalities along the tracts of the olfactory cortex were among the most frequent neuroimaging abnormalities reported in patients with COVID-19 [8, 10, 23, 29, 32, 34, 36–39, 41–43, 45, 47–49, 52, 54, 55, 57, 58, 60, 62–64, 66–70, 75, 79, 81–86, 95, 96, 98–101, 103, 107–109, 113, 115]. This finding corroborates the observations of previous studies [13, 127, 128]. Other neuroimaging findings included ischaemic or haemorrhagic stroke [37, 56, 81], cerebral venous sinus thrombosis [21, 88, 105], and acute or subacute infarction [54, 68, 107]. Of note, Ntaios et al. demonstrated that patients with ischaemic stroke related to COVID-19 had worse functional outcomes and higher mortality than patients with ischaemic stroke and without COVID-19 [129].

Spinal cord involvement in the acute phase presented unremarkable features, especially in non-critical patients. Comprehensive MRI studies have reported hyperintense enhancement with spinal cord involvement across several cases of transverse myelitis, meningoencephalitis, and other acute inflammatory changes, characterised by oedema of the central cord and paraspinal musculature. This finding is consistent with a recent review of case studies of spinal involvement in COVID-19 infection [150].

### Neuroanatomical Changes in the Chronic Phase of the Disease

In patients with long COVID-19, the basal ganglia and thalamus were predominately implicated, with hypometabolism reported regionally across the frontal, parietal and occipital lobes and related impairments emanating from the temporal lobe. Notably, neuroimaging investigations have evidenced spinal cord degenerative changes, demyelinated plaques, and spinal lesions observed among patients with persistent symptoms or long COVID [108, 147]. The persistence of symptomologies of neurological relevance in long COVID-19 patients relate to residual genetic material (i.e., ribonucleic acid) of SARS-COV‑2 in the central nervous system after the acute phase of the disease, which potentially results in neuronal loss and/or a delayed restoration of neuroanatomy [130]. Additionally, systemic inflammation following the active acute phase of the COVID-19 infection may potentially cause system level endotheliitis and consequently disrupt the blood–brain barrier [131, 132]. Moreover, it is known that systemic hyperinflammation is a leading cause of neurodegeneration and cognitive decline following regional brain alterations [133, 134]. In relation to the pathophysiology and underlying mechanism(s) of long COVID-19, Baig [135] suggested that oxidative stress and inflammation leads to weakened immunological response and incomplete virus eradication [3, 135], which explains the relative hypometabolism reported across regional cortices following clinical recovery from acute COVID-19.

It is somewhat surprising that out of 101 included studies, only 5 studies (4.95%) reported on long COVID-19 patients with persisting brain changes postrecovery. To the best of our knowledge, our review revealed a large clinical gap related to the lack of literature on long COVID-19 patients.

Locally, incidental neuroimaging changes were uncovered in patients with concurrent, recent or previous COVID-19 infection. These included acute ischaemic infarcts, presumed microhaemorrhages, atrophic changes, and white matter foci (supplement 1). While these changes were anecdotal and cannot be proven as a direct or indirect result of SARS-Cov‑2 infection, clinicians globally are likely to have seen similar nonspecific topographical changes on neuroimaging in conjunction with COVID-19, in turn complicating both accurate diagnosis and subsequent patient management. Further research to compare the incidence of these neuroimaging changes, and similar, in patients affected by COVID-19 (acute and chronic) and those unaffected would add important insight to this discussion.

### Strengths and Limitations

This review used an extensive search strategy to collect relevant available evidence on associated abnormal brain and spinal regions on neuroimaging following COVID-19 infection, highlighting the clinicoradiologic findings based on neurological symptoms and neuroimaging modalities. Similarly, the study followed a rigorous method for article screening, and data extraction, and employed a standardised risk of bias assessment tool appropriate to the study designs that influenced the discussions and recommendations.

This study has some limitations that need to be considered. Firstly, by only including studies published in English, we may have excluded some valuable studies published in other languages. Secondly, in relation to the quality of the included studies, a large percentage had a high risk of bias in participant selection and blinding of outcome assessment, a low risk of bias due to incomplete outcome data, and an unclear risk of selective outcome reporting; however, the geographic distribution of the included studies is diverse and represent generalisable demographics. Thirdly, the quality of our included studies did not allow for a meta-analysis due to disparity in the findings for acute and long COVID-19 studies and the heterogeneity of the methodological designs of the included studies. Our findings should therefore be interpreted with caution considering the relatively low number of studies relating to long COVID-19.

### Implications for Future Research, Policy, and Practice

A plethora of studies highlighting neuroanatomical changes following acute COVID-19, albeit little evidence is currently available in relation to long COVID-19 patients. The lack of studies on regional neuroanatomical changes in long COVID-19 requires further research to bridge this gap. Recent studies have demonstrated the need to focus a new lens on the COVID-19 pandemic and pay attention to long-term impacts of SARS-Cov‑2 infection of the brain [11, 136] in accordance with the WHO action plan to better understand the disease [137]. Drawing upon the Global Health 50/50, the African Population and Health Research Centre and the International Centre for Research on Women Statement on Global Tracking of COVID-19 [138], an in-depth understanding of how biological sex affects COVID-19 will have important implications for clinical management and mitigating strategies for this disease.

Further longitudinal studies with longer follow-ups are needed to evaluate clinical consequences (e.g., initial infection vs. reinfection, prevaccination vs. postvaccination COVID infection) and neuroabnormalities [139, 140] as well as other regional implications of neurological relevance (e.g., spinal involvement). Studies have reported the increasing adoption of machine learning techniques in the medical field due to their high accuracy [141, 142]. Therefore, future work should include machine learning algorithms to predict the impact of COVID-19 on affected brain and spinal regions [143]. As research in this area increases, future studies will be able to draw more complete neuroanatomical conclusions in patients with both acute and long COVID-19.

## Conclusion

This systematic review presents evidence relating to the frequency of occurrence and topographical distribution of neuroanatomical abnormalities seen on brain and spinal imaging following COVID-19 infection across the acute and longer term phases of the disease. These findings contribute to our understanding of the acute and chronic effects of the virus on the brain and has the potential to inform acute and long-term treatment and neurorehabilitation decisions.

### Supplementary Information

S1 Table. Summary characteristics of the included studies.

S2 Table. Risk of bias assessment tool for non-randomized studies (RoBANS)
